# Irisin attenuates myocardial ischemia/reperfusion‐induced cardiac dysfunction by regulating ER‐mitochondria interaction through a mitochondrial ubiquitin ligase‐dependent mechanism

**DOI:** 10.1002/ctm2.166

**Published:** 2020-09-14

**Authors:** Linhe Lu, Jipeng Ma, Jiayou Tang, Yang Liu, Qijun Zheng, Shasha Chen, Erhe Gao, Jun Ren, Lifang Yang, Jian Yang

**Affiliations:** ^1^ Department of Cardiovascular Surgery Xijing Hospital Air Force Medical University Xi'an China; ^2^ Department of Cardiovascular Surgery Shenzhen People's Hospital Second Clinical Medical College Jinan University Shenzhen China; ^3^ Center for Translational Medicine Lewis Katz School of Medicine at Temple University. Philadelphia Pennsylvania USA; ^4^ Center for Cardiovascular Research and Alternative Medicine University of Wyoming Laramie Wyoming USA; ^5^ Department of Anesthesiology Xi'an Children's Hospital Xi'an China

**Keywords:** apoptosis, endoplasmic reticulum stress, irisin (FNDC5), mitochondrial ubiquitin ligase (MITOL), myocardial ischemia/reperfusion (MI/R), reactive oxygen species (ROS)

## Abstract

**Background:**

Myocardial ischemia/reperfusion (MI/R) injury imposes devastating cardiovascular sequelae in particular cardiac dysfunction as a result of restored blood flow. However, the mechanism behind MI/R injury remains elusive. Mitochondrial ubiquitin ligase (MITOL/MARCH5) is localized at the mitochondria‐ER contact site and may be activated in response to a variety of pathophysiological processes, such as apoptosis, mitochondrial injury, ER stress, hypoxia, and reactive oxygen species (ROS) generation. Irisin as a cleaved product of fibronectin type III domain‐containing protein 5 (FNDC5) displays cardioprotection in diverse cardiac diseases.

**Methods:**

This study was designed to examine the role of irisin and MITOL in MI/R injury. Male C57BL/6J mice (8‐10‐week‐old) were administered adenovirus MITOL shRNA through intracardiac injection followed by MI/R surgery through ligation and release the slipknot of cardiac left anterior descending coronary artery.

**Results:**

Our results showed that irisin improved myocardial function in the face of MI/R injury as evidenced by reduced myocardial infarct size, apoptotic rate, serum lactate dehydrogenase (LDH), ROS generation, and malondialdehyde (MDA) levels as well as lessened ER stress injury. Moreover, our results indicated that protective role of irisin was mediated by upregulation of MITOL. Irisin also protected H9c2 cells against simulated I/R through negating ER stress, apoptosis, ROS and MDA levels, as well as facilitating superoxide dismutase (SOD) by way of elevated MITOL expression.

**Conclusions:**

To this end, our data favored that irisin pretreatment protects against MI/R injury, ER stress, ROS production, and mitochondrial homeostasis through upregulation of MITOL. These findings depicted the therapeutic potential of irisin and MITOL in the management of MI/R injury in patients with ST‐segment elevation.

## Graphical abstract

Irisin improves cardiac function against myocardial ischemia/reperfusion (MI/R) injury, decreases apoptosis and myocardial infarct size following MI/R injury, alleviates reactive oxygen species (ROS) production following MI/R injury, and protects against MI/R injury, ER stress, and mitochondrial homeostasis through upregulation of MITOL

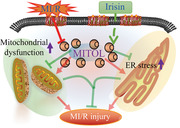

## 1 BACKGROUND

Acute myocardial infarction is one of the acute coronary syndromes with high mortality and morbidity worldwide.^1^ Despite the recent advance in medical care to restore blood flow through timely thrombolysis, coronary artery bypass grafting, and percutaneous coronary intervention (PCI), irreversible cardiomyocyte death, decreased systolic function, and eventually heart failure still develop.^2^, ^3^ This pathological process is commonly known as myocardial ischemia/reperfusion (MI/R) injury seen in patients with acute ST‐segment elevation myocardial infarction (STEMI).^4^ Presence of MI/R often leads to inflammation, intracellular Ca^2+^ overload, oxidative stress, endoplasmic reticulum (ER) stress, and ultimately irreversible cell death involving apoptosis and necrosis.^5^, ^6^, ^7^, ^8^, ^9^ However, precise mechanisms of ischemia/reperfusion (I/R) injury have not been fully elucidated. It is essential to clarify the underlying mechanisms in an effort to engage effective and safe therapeutic strategies to reduce infarct size of myocardium in I/R injury and promote cardiac capacity to resist future injuries. Although various medications have been reported in an effort to combat MI/R injury,^5^, ^10^ pharmacotherapy still remains dismal for the management of MI/R injury.

Irisin is a newly identified proliferator‐activated receptor‐γ coactivator‐1α‐dependent myokine released into circulation during exercise.^11^ Irisin is a cleaved product of fibronectin type III domain‐containing protein 5 (FNDC5) secreted by myocardium and skeletal muscle, and was initially considered an exercise‐provoked hormone for energy expenditure and browning of white adipose tissues.^11^, ^12^ Recent studies have indicated a therapeutic potential for irisin in cardiovascular diseases by facilitating cardiac function and myocardial repair in infarcted hearts.^13^ Wang and colleagues found that irisin protected against MI/R and hypoxia/reoxygenation injury, in association with improved mitochondrial function.^14^, ^15^, ^16^ More evidence has indicated an important role for irisin in regulating ER stress and apoptosis.^17^, ^18^, ^19^ In particular, irisin has been demonstrated to alleviate cardiac injuries through machineries including mTOR/AMPK/ULK1, autophagy induction, inhibition of ROS/TGFβ1/Smad2/3 signaling axis, and SOD2‐dependent mitochondrial regulation.^20^, ^21^, ^22^, ^23^

The ER is the primary site for the synthesis, folding, and processing of proteins, and a cadre of essential cellular functions such as Ca^2+^ storage/release and lipid synthesis.^24^ Disturbed ER homeostasis leads to the accumulation of misfolded and unfolded proteins within the ER, a process known as ER stress.^25^ ER stress is activated to combat the load of newly synthesized proteins by degrading misfolded proteins and shutting down protein translocation in physiological conditions. Ample evidence has consolidated a role for ER stress in the pathological process of cardiovascular diseases.^26^ For example, cardiac performance decreased followed by ER stress activation during myocardial infarction or ischemia injuries, resulting in heart failure.^27^ The inositol requiring kinase enzyme 1α (IRE1α) is the most conserved ER‐resident unfolded protein response (UPR) regulator to re‐establish cell homeostasis under mild ER stress, although excessive or sustained ER stress triggers cell death.^28^ Bischoff and colleagues reported upregulated IRE1α in cardiomyocytes subjected to hypoxia while reducing IRE1α activity offered cardioprotection.^29^ To this end, attenuation of ER stress through IRE1α might be a potential strategy for treating MI/R injury.^30^, ^31^

Cardiomyocytes are heavily enriched with mitochondria, which constitute important cellular functions including ATP synthesis, Ca^2+^ buffering and apoptosis.^32^ Mitochondria also serve as the major site for reactive oxygen species (ROS) production following MI/R injury.^33^ Accumulation of ROS leads to mitochondrial injury and eventually cardiomyocyte apoptosis and heart disease.^34^ Among multiple components governing mitochondrial integrity and dynamics, the mitochondrial ubiquitin ligase MITOL/MARCH5 participates in the regulation of mitochondrial dynamics against hypoxic and ROS generation.^35^, ^36^, ^37^ Diverse studies have demonstrated an anti‐apoptotic role for MITOL in cardiomyocytes in addition to regulation of the interaction at the mitochondria and ER contact site.^28^, ^38^, ^39^ Previous study has noted disturbed regulation IRE1α and ER stress with inhibited MITOL expression.^28^ However, whether ER stress and MITOL signaling participates in irisin‐elicited cardiac protection against MI/R, if any, remain unclear. Therefore, this study was designed to elucidate the effect of irisin on cardiac function following MI/R injury and the mechanism involved with a focus on ER stress and MITOL signaling.

## 2 MATERIALS AND METHODS

### 2.1 Ethics statement

All male C57BL/6J mice (8‐10‐week‐old) involved in this study were provided by the Laboratory Animal Center of Air Force Medical University. All experimental procedures were performed in compliance with the 2011 Guide for the Care and Use of Laboratory Animals, and the study protocol was approved by Air Force Medical University Experimental Animal Research Committee. All animals were fed with food and water ad libitum and were kept at 23‐25°C under a 12:12 h light‐and‐dark cycle.

### 2.2 Reagents

4′,6‐Diamino‐2‐phenylindole (DAPI) was purchased from Sigma‐Aldrich (St. Louis, MO, USA). Evans blue (EB) and triphenyltetrazolium chloride (TTC) were obtained from Solarbio Life Sciences (Beijing, China). The terminal deoxynucleotidyltransferase‐mediated dUTP nick end labeling (TUNEL) assay kit was purchased from Roche (Mannheim, Germany). GAPDH antibody was purchased from Santa Cruz Biotechnology (Dallas, TX, USA). MITOL and Irisin/FNDC5 antibody were purchased from Abcam (Cambridge, UK). Goat anti‐rabbit (ZB‐2301), goat anti‐mouse (ZB‐2305), and rabbit anti‐goat (ZB‐2306) secondary antibodies were purchased from the ZSGB‐Bio (Beijing, China). Recombinant irisin protein was purchased from Cloud Clone (Wuhan, China). MITOL shRNA adenovirus was purchased from GeneChem (Shanghai, China). MITOL siRNA was purchased from Santa Cruz Biotechnology (CA, USA).

### 2.3 Intracardiac injection and in vivo MI/R injury protocol

Recombinant irisin was intraperitoneally injected with 0.5 mg/kg body weight every day for 1 week and at the time point of 30 min before MI/R surgery.^40^ Intracardiac injection of MITOL shRNA adenovirus was administered three days before MI/R injury. MITOL shRNA adenovirus went through in three evenly spaced injections into the left ventricle (7 μL each at a concentration of 1 × 10^11^ PFU/mL). The MI/R surgery was performed as previously described.^41^ Mice were anesthetized by inhalation of an isoflurane‐oxygen mixture (2‐3%) and maintained anesthesia with isoflurane of 1.5‐2%. In brief, myocardial ischemia was performed by temporarily exteriorizing the heart from the fourth intercostal space and then placing a slipknot around the left anterior descending coronary artery of the heart with a 6‐0 silk suture. Thirty minutes after the ischemia, the slipknot was released. For protein analysis, hearts were reperfused for 4 h before harvest. For cardiac functional measurements, TTC staining and TUNEL staining, mice hearts were reperfused for 24 h. In the sham‐operated mice, the suture was left untied. Blood was collected and centrifuged for 10 min at 3000 × *g* to separate the serum.

### 2.4 In vivo experimental protocol

The mice were randomly divided into four groups in the first experiment (n = 20‐25 for each group): (a) Sham group: mice underwent a sham operation and received normal saline. (b) Sham‐Irisin group: mice were treated with recombinant irisin and the sham operation was received with saline. (c) MI/R group: mice underwent MI/R surgery and were treated by normal saline. (d) MI/R‐Irisin group: mice were administrated with recombinant irisin for 1 week and at the time point of 30 min before operation and then MI/R surgery was performed.

In the second experiment, mice were randomly assigned into four groups (n = 20‐25 for each group). (a) MI/R‐Ad‐ctrl group: mice received normal saline and underwent intracardiac injection with empty adenovirus. Then MI/R surgery was performed 3 days after the injection. (b) MI/R‐Irisin group: mice were treated with recombinant irisin and were intracardiacally injected with Ad‐ctrl adenovirus. Then MI/R surgery was performed. (c) MI/R‐MITOL‐shRNA group: mice were pretreated with a MITOL shRNA adenovirus via intracardiac injection at 3 evenly space site of LV (7 μL each at a concentration of 1 × 10^11^ PFU/mL) and MI/R surgery was performed 3 days after the injection. (d) MI/R‐Irisin‐MITOL‐shRNA group: mice were pretreated with recombinant irisin and were intracardiacally injected with a MITOL shRNA adenovirus. Then MI/R surgery was performed 3 days after the adenovirus injection.

### 2.5 Echocardiographic assessment

For cardiac function evaluation, echocardiography (2‐dimensional and M‐mode) was conducted with a VEVO 2100 high‐resolution in vivo imaging system (VisualSonics, Toronto, ON, Canada) as previously described.^42^ Echocardiography was performed while the animal was anaesthetized by inhalation of 2‐4% isoflurane with maintenance of stable body temperature at around 37°C. The left ventricular ejection fraction (LVEF) and left ventricular fractional shortening (LVFS) were obtained 1 day after performing the MI/R procedure.

### 2.6 Evans blue /TTC staining

After echocardiographic measurements, the mouse was anesthetized by inhalation of 2‐4% isoflurane and the ligation of the coronary artery was performed at the place where it was previously ligated in MI/R surgery. Subsequently, Evans Blue solution was injected into coronary artery system of the hearts. After frozen on the dry ice, the whole heart was cut into four pieces. Then the cardiac tissue was stained by TTC solution at 37°C for 30 min for Evans blue /TTC staining double staining and the infarct size was determined using the Image‐Pro Plus software (Media Cybernetics, Rockville, MD, USA) and the result was expressed by infarct area/area at risk × 100%.

### 2.7 Cell viability assay

The Cell Counting Kit‐8 (CCK‐8, Dojindo, Kumamoto, Japan) assay was used to determine the effect of irisin on H9c2 cell viability following the manufacturer's instructions. H9c2 cells were seeded in a 96‐well culture plate. Recombinant irisin (FNDC5, Cloud Clone Corp, Wuhan, China) was reconstituted in buffer (20 mM Tris, 150 mM NaCl pH 8.0 diluted to a concentration of 1 μg/mL) as described in the manufacturer's instruction manual and then diluted to the following concentrations: 1, 2, 5, 10, 20, and 50. Vehicle control cells were cultured in DMEM (Hyclone, Logan, UT, USA) supplemented with 0.2% buffer as described above. Five duplicate wells were repeated for each group.

### 2.8 Cell culture and simulated ischemia/reperfusion (SI/R) injury in vitro

H9c2 cells were cultured in DMEM medium supplemented with 10% FBS. The H9c2 cells were cultured and used for the following in vitro experiments.

To simulate I/R injury in vitro, H9c2 cells were cultured in an ischemic buffer (4.007 g NaCl, 0.074 g CaCl_2_·2H_2_O, 0.59 g KCl, 0.826 g deoxyglucose, 0.093 g sodium dithionate, 0.475 g HEPES, and 1.12 g Lactate pH 6.5 in 500 mL deionized water) for 50 min in a cell culture incubator. Reperfusion was performed by exposing the cells to serum‐free DMEM with or without irisin for 4 h. H9c2 cells were transfected with or without the MITOL siRNA strictly following the manufacturer's instructions, then randomly divided into the following groups: (a) control (Con): cells were treated with serum‐free DMEM; (b) MITOL siRNA (MITOL‐siRNA): cells were pretreated with MITOL siRNA for 24 h; (c) hypoxia/reperfusion (SI/R): cells were placed in an ischemic buffer for 50 min and then returned to serum‐free DMEM for 4 h; (d) hypoxia/reperfusion+irisin (SI/R‐Irisin): cells were pretreated with irisin for 4 h and then placed in an ischemic buffer for 50 mins followed by incubation in serum‐free DMEM for 4 h; (e) hypoxia/reperfusion‐MITOL siRNA (SI/R‐Irisin‐MITOL‐siRNA): cells were pretreated with MITOL siRNA for 24 h and irisin for 4 h then placed in an ischemic buffer for 50 min followed by incubation in serum‐free DMEM for 4 h.

### 2.9 Intracellular ROS measurement of H9c2 cells by DCFH‐DA

The intracellular ROS level in H9c2 cells was measured with 2′,7′‐dichlorodi‐hydrofluorescein diacetate (DCFH‐DA) (Nanjing jiancheng, Nanjing, China). Briefly, H9c2 cells (10^4^ cells/mL) were cultured with DMEM medium for 24 h and then SI/R‐induced injury was established in the presence or absence of MITOL siRNA or irisin. The cells were then incubated with 10 μM DCFH‐DA in DMEM medium without FBS at 37°C for 1 h. Afterward, the cells were washed three times with PBS and the fluorescent images were observed under an Olympus Fluoview FV1000 microscope (Olympus, Tokyo, Japan). The fluorescence intensity of the images was quantified by using Image J software (National Institutes of Health, Bethesda, MD).

### 2.10 Serum LDH concentration, myocardial MDA and GSH‐Px activity, and myocardial SOD activity determination

Blood samples were obtained from the carotid artery 24 h after MI/R surgery and stored in a non‐anticoagulant tube at 25°C for 30 min. The samples were then centrifuged at 3000 × *g* for 10 min and the serum was collected for the determination of lactate dehydrogenase (LDH, Jiancheng, Nanjing, Jiangsu, China) concentration.

For measuring malondialdehyde (MDA, Jiancheng, Nanjing, Jiangsu, China) content, glutathione peroxidase (GSH‐Px, Jiancheng, Nanjing, Jiangsu, China), and superoxide dismutase (SOD, Jiancheng, Nanjing, Jiangsu, China) activities of myocardium, cardiac total proteins were obtained and the assay was performed according to the manufacturer's instructions by using a SpectraMax M5 microplate reader (Molecular Devices, CA, USA).

### 2.11 Terminal deoxynucleotidyl transferase dUTP nick end labeling (TUNEL) assay

TUNEL analysis using an in situ cell death detection kit (Roche Molecular Biochemicals, Mannheim, Germany) was performed to determine the myocardial apoptosis and cellular apoptosis following the manufacturer's instructions. In brief, nuclei were visualized by DAPI staining after apoptotic cells were labeled by TUNEL reaction mixture. The samples were examined under an Olympus Fluoview FV1000 microscope (Olympus, Japan) and the results are presented as an apoptotic rate (×100%).

### 2.12 Western blotting

The left ventricular tissue of hearts and H9c2 cells were harvested after diverse treatments and prepared for western blotting. The separation of cytosol and mitochondrial protein fraction from cardiac tissues and H9c2 cells were obtained by using a mitochondria isolation kit (Beyotime, Shanghai, China) according to the manufacture's instruction. After separating the protein samples by SDS‐PAGE, the proteins were transferred onto a PVDF membrane (Millipore, Billerica, MA, USA) and blocked with 5% nonfat milk in TBST. The membrane was then incubated with primary antibodies at 4°C overnight. The antibodies against Bip/GRP78 (1: 1,000), IRE1α (1: 1,000), Total OXPHOS, irisin, and p‐ IRE1α (1: 1,000) were purchased from Abcam while the antibodies against XBP‐1s (1:1000), ATF4 (1:1000), CHOP (1:1000), Caspase 3 (1:1000), Bax (1:1000), Bcl‐2 (1:1000), Cyto C (1:1000), and GAPDH (1:5000) were purchased from Cell Signaling Technology. After wash with TBST, the membranes were incubated with HRP‐conjugated second antibodies for 1.5‐2 h at RT. Then the proteins were visualized using chemiluminescent reagents (Millipore, Billerica, MA, USA) under ChemiDoc Imaging System (Bio‐Rad Laboratories, Hercules, CA, USA) and the densities of the bands were quantified by Image Lab software (Bio‐Rad Laboratories, Hercules, CA, USA). GAPDH was used as an internal reference.

### 2.13 Statistical analysis

All numerical data are expressed as mean ± standard error of the mean (SEM). Data were analyzed by the GraphPad Prism software version 8.0 (GraphPad Software, Inc., San Diego, CA). Statistical analysis of differences between the groups was performed by two‐way ANOVA followed with multiple comparisons by post hoc Tukey's test. The differences at *P*‐values of .05 or less were considered statistically significant.

## 3 RESULTS

### 3.1 Irisin improves cardiac function and decreases apoptotic index, myocardial infarct size, and serum LDH level following MI/R injury

**FIGURE 1:**
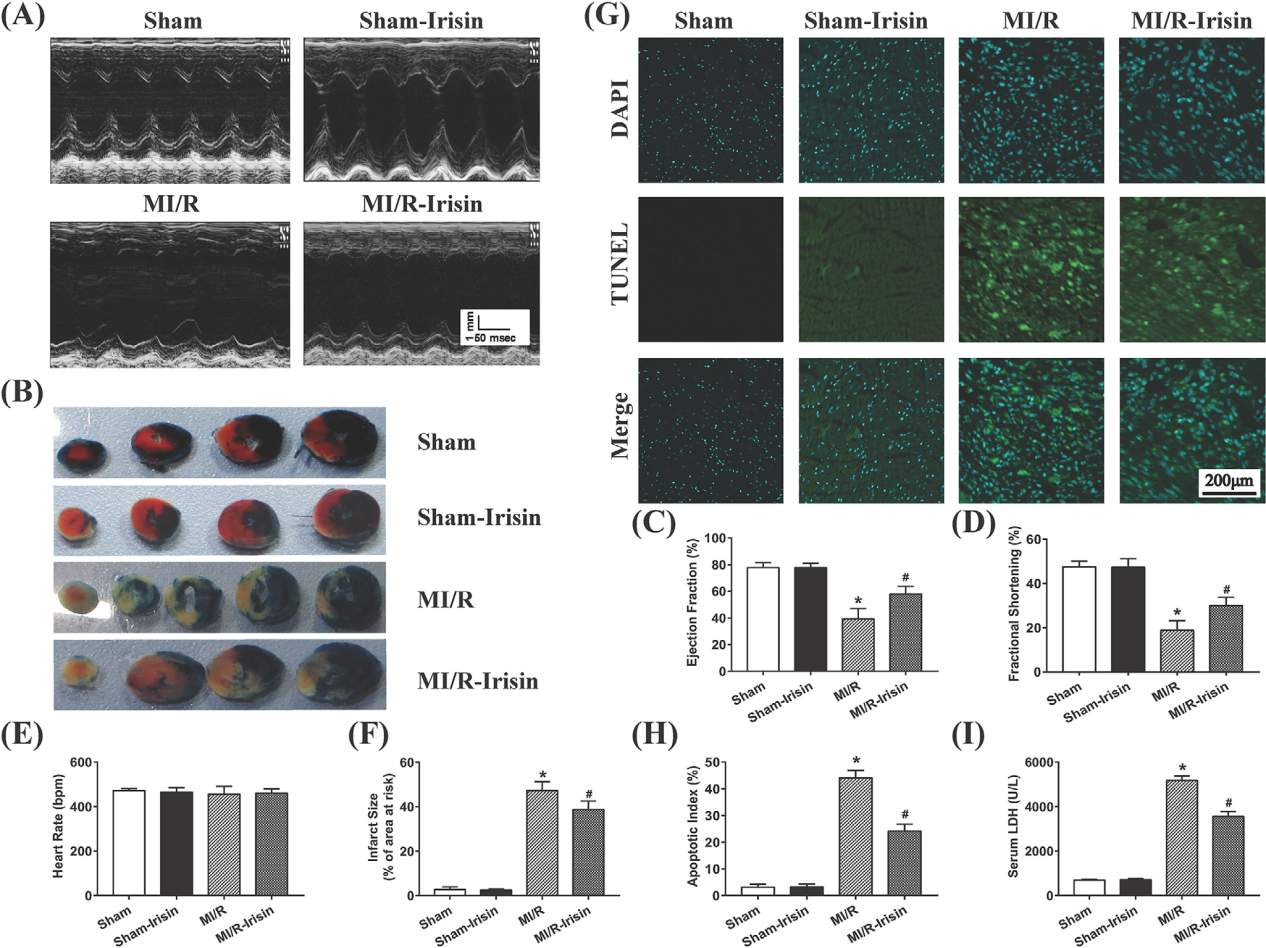
The effects of irisin treatment on heart function, infarct size, apoptotic index, and LDH release following MI/R injury. A, Representative M‐mode images of echocardiography under MI/R injury or sham operation with or without irisin treatment. B, Representative images of cardiac sections by Evans blue and TTC double staining. The blue‐stained portion indicates the non‐ischemic, normal region. The red‐stained portion indicates the ischemic/reperfused but non‐infarcted region. The non‐stained portion indicates the infarcted region. C, Left ventricular ejection fraction (LVEF). D, Left ventricular fractional shortening (LVFS). E, Heart rate. F, Myocardial infarct size was expressed as percentage of area at risk (AAR). G, Representative images of apoptotic cardiomyocytes. TUNEL: green fluorescence represents TUNEL‐positive nuclei. DAPI: blue fluorescence represents total cardiomyocyte nuclei. H, Cell apoptosis was presented as the apoptotic index (×100%). I, Serum LDH level. Sham: mice underwent sham operation. Sham‐Irisin: mice were pretreated with recombinant irisin underwent sham operation. MI/R: MI/R surgery was performed in this group. MI/R‐Irisin: mice were pretreated with recombinant irisin then subjected into MI/R surgery. The results are presented as mean ± SEM, n = 3‐6 in each group. ^*^ *P* < 0.05 versus Sham group, ^#^ *P* < 0.05 versus the MI/R group

Echocardiographic data suggested that MI/R injury overtly decreased LVEF and LVFS, the effect of which was alleviated by pretreatment of irisin with little effect from irisin itself (Figure 1A,C,D). MI/R increased myocardial infarct size (Figure 1B,F). Furthermore, MI/R injury significantly promoted cardiomyocyte apoptosis as evidenced by increased myocardial apoptotic index (Figure 1G,H) and serum LDH level (Figure 1I). Although irisin treatment itself did not elicit any notable effect on myocardial apoptosis, infarction, and serum LDH levels, it significantly alleviated MI/R‐induced rises in apoptotic index, myocardial infarction area, and serum LDH level. These results indicate that irisin overtly improved cardiac function and ameliorated myocardial apoptosis in the face of MI/R injury.

### 3.2 Irisin increases levels of MITOL and ETC complexes and alleviates ER stress induced by MI/R injury

**FIGURE 2:**
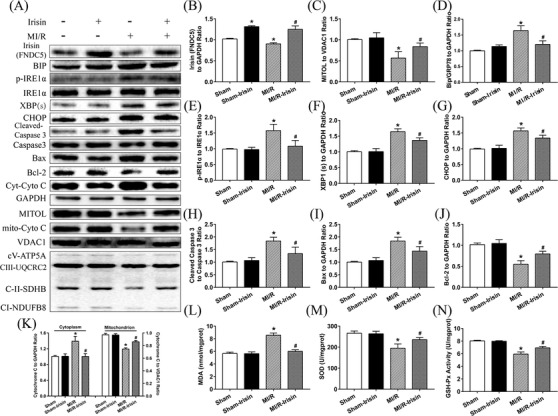
Irisin treatment increased MITOL expression, reduced cardiac ER stress, decreased myocardial apoptosis and MDA levels, and increased SOD and GSH‐Px activities in vivo following MI/R injury. A, Representative protein images by western blot from different groups. B, Irisin levels in cardiac tissues. C, Expression of MITOL. D‐G, Expressions of ER stress related proteins. H‐K, Expression of apoptotic related proteins. L, Myocardial MDA content. M, Myocardial SOD activity. N, Myocardial GSH‐Px activity. Sham: mice underwent sham operation. Sham‐Irisin: mice were pretreated with recombinant irisin underwent sham operation. MI/R: MI/R surgery was performed in this group. MI/R‐Irisin: mice were pretreated with recombinant irisin then subjected into MI/R surgery. The results are presented as mean ± SEM, n = 3‐4 in each group. ^*^ *P* < 0.05 versus the Sham group, ^#^ *P* < 0.05 versus the MI/R group

Western blot results revealed that expression of MITOL was downregulated while levels of ER stress protein phosphorylated IRE1α (p‐IRE1α) were overtly increased following MI/R injury compared with the Sham group, the effects of which were reversed by irisin pretreatment, with little effect from irisin itself. MI/R significantly reduced the expressions of mitochondrial Electron Transport Chain (ETC) complexes (CV‐ATP5A, CIII‐UQCRC2, CIV‐MTCO1, CII‐SDHB, CI‐NDUFB8) while treatment of irisin upregulated the expressions of mitochondrial ETC complexes compared to MI/R group as shown in Figure 2A. Further examination of ER stress protein markers revealed that MI/R injury markedly upregulated levels of Bip (GRP78) and XBP1(s), the effect of which was negated by irisin treatment. Given that sustained activation of ER stress promotes the shift from cell survival to apoptosis through IRE1 signaling,^43^ we determined the expression of CHOP that was significantly increased after MI/R injury, although such effect was reversed by irisin pretreatment (Figure 2A‐G).

### 3.3 Irisin upregulates GSH‐Px and SOD activities, and decreases MDA level and myocardial apoptotic proteins following MI/R injury

As shown in Figure 2H‐N, MI/R procedure significantly upregulated levels of cleaved Caspase 3, cytosolic cytochrome C, and Bax while decreasing that of Bcl‐2 and mito‐Cyto C, the effect of which was obliterated by irisin treatment with little effect from irisin itself. Moreover, MDA level was significantly elevated although activity of SOD and glutathione peroxidase (GSH‐Px) was reduced in myocardium following MI/R injury, the effect of which was reversed by irisin treatment. These data suggest that irisin alleviates MI/R‐induced apoptosis possibly related to regulation of redox balance.

### 3.4 MITOL mediates irisin‐offered cardioprotection against MI/R injury

**FIGURE 3:**
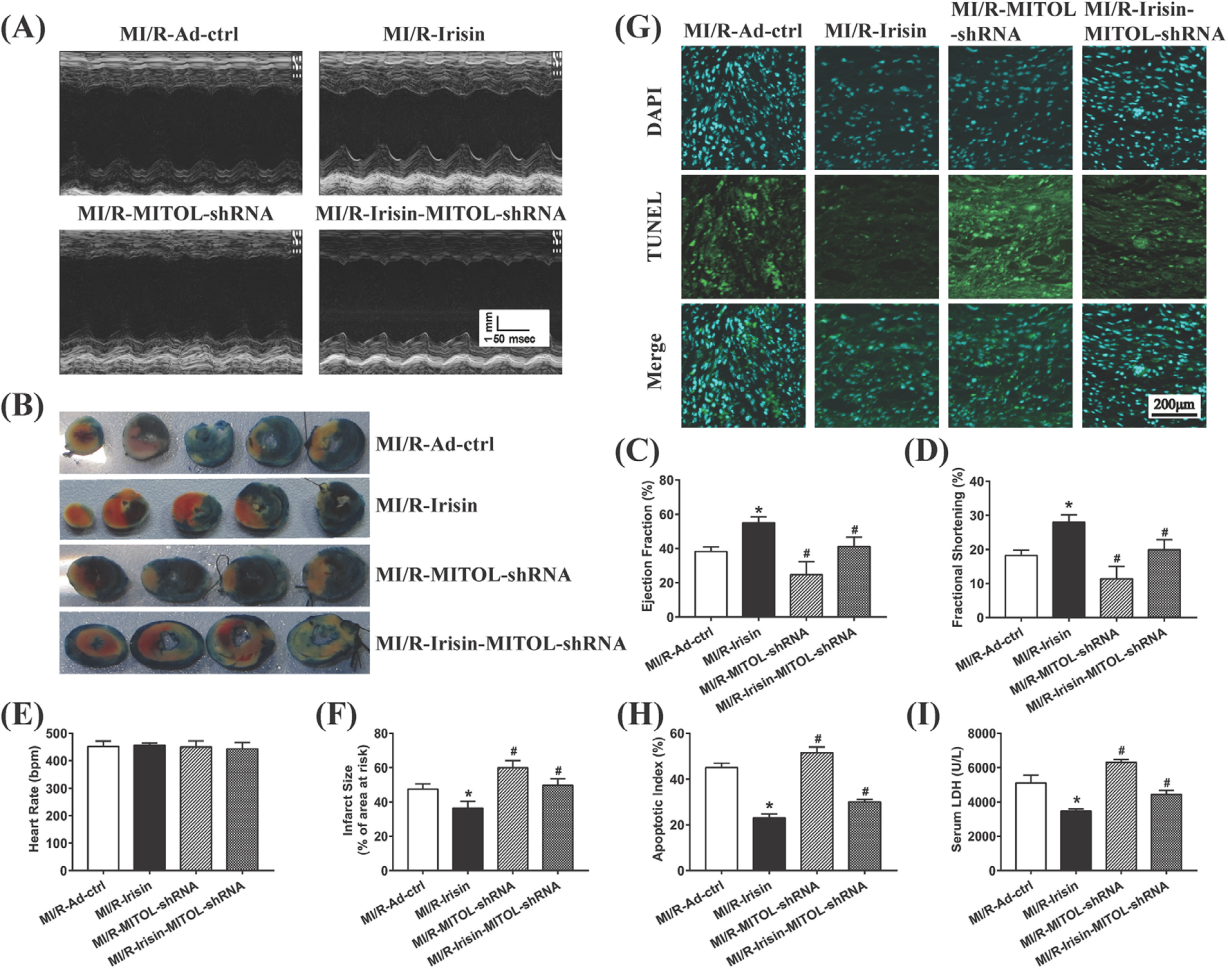
The effects of MITOL inhibition on heart function, infarct size, apoptotic index, and LDH release following MI/R injury and irisin treatment. A, Representative M‐mode images of echocardiography. B, Representative images of cardiac sections by Evans blue and TTC double staining. The blue‐stained portion indicates the non‐ischemic, normal region. The red‐stained portion indicates the ischemic/reperfused but non‐infarcted region. The non‐stained portion indicates the infarcted region. C, Left ventricular ejection fraction (LVEF). D, Left ventricular fractional shortening (LVFS). E, Heart rate. F, Myocardial infarct size was expressed as a percentage of area at risk (AAR). G, Representative images of apoptotic cardiomyocytes. TUNEL: green fluorescence represents TUNEL‐positive nuclei. DAPI: blue fluorescence represents total nuclei of cardiomyocytes. H, Cell apoptosis was presented as the apoptotic index (×100%). I, Serum LDH level. MI/R‐Ad‐ctrl: mice injected Ad‐ctrl adenovirus underwent MI/R surgery. MI/R‐Irisin: mice were pretreated with irisin then underwent MI/R surgery with Ad‐ctrl adenovirus injection. MI/R‐MITOL‐shRNA: mice underwent MI/R surgery with MITOL‐shRNA adenovirus injection. MI/R‐Irisin‐MITOL‐shRNA: mice were pretreated with irisin then underwent MI/R surgery with MITOL‐shRNA adenovirus injection. The results are presented as mean ± SEM, n = 3‐6 in each group. ^*^ *P* < 0.05 versus the MI/R group, ^#^ *P* < 0.05 versus the MI/R‐Irisin group

To determine the possible role of MITOL in irisin‐offered cardioprotection against MI/R injury, mice were treated with adenovirus MITOL shRNA and recombinant irisin prior to MI/R procedure. Ad‐MITOL‐shRNA treatment nullified irisin‐induced protection against MI/R injury (Figure 3A,C,D). In addition, Ad‐MITOL‐shRNA treatment increased infarct size (Figure 3B,F), apoptotic index (Figure 3G,H), and serum LDH level (Figure 3I). These observations indicate a vital role for MITOL in irisin‐mediated cardioprotective effect against MI/R injury.

### 3.5 MITOL participates in irisin‐mediated attenuation of ER stress‐induced cardiac dysfunction post MI/R injury

**FIGURE 4:**
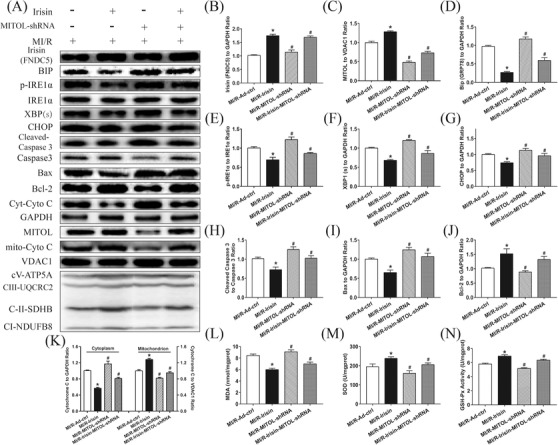
MITOL inhibition impaired the protective role of irisin on cardiac ER stress, myocardial apoptosis and MDA contents, and SOD and GSH‐Px activities induced by MI/R in vivo. A, Representative protein images by western blot. B, The cardiac irisin levels following irisin treatment and MITOL shRNA adenovirus injection. C, Expression of MITOL. D‐G, Expressions of ER stress related proteins. H‐K, Expression of apoptotic related proteins. L, Myocardial MDA content. M, Myocardial SOD activity. N, Myocardial GSH‐Px activity. MI/R‐Ad‐ctrl: mice injected Ad‐ctrl adenovirus underwent MI/R surgery. MI/R‐Irisin: mice were pretreated with irisin then underwent MI/R surgery with Ad‐ctrl adenovirus injection. MI/R‐MITOL‐shRNA: mice underwent MI/R surgery with MITOL‐shRNA adenovirus injection. MI/R‐Irisin‐MITOL‐shRNA: mice were pretreated with irisin then underwent MI/R surgery with MITOL‐shRNA adenovirus injection. The results are presented as mean ± SEM, n = 3‐4 in each group. ^*^ *P* < 0.05 versus the MI/R group, ^#^ *P* < 0.05 versus the MI/R‐Irisin group

As shown in Figure 4A‐G, pretreatment with irisin significantly decreased the expressions of Bip, p‐ IRE1α, XBP1(s), CHOP, and mitochondrial ETC complexes compared to IR‐Ad‐ctrl group while MITOL shRNA treatment weakened the reduction of irisin on ER stress and mitochondrial ETC complexes following MI/R injury. Furthermore, inhibition of MITOL promoted ER stress activation. These results suggest that administration of irisin alleviates MITOL inhibition‐triggered ER stress following MI/R injury.

### 3.6 MITOL participates in irisin‐mediated decrease in myocardial apoptosis and MDA levels, and upregulation of SOD and GSH‐Px activities induced by MI/R injury

As shown in Figure 4H‐N, irisin treatment overtly reduced the apoptotic proteins cleaved Caspase 3, cytosolic Cytochrome C and Bax expressions but upregulated the anti‐apoptotic protein Bcl‐2 expression and the expression of mito‐Cyto C, the effect of which was impaired by intracardiac injection of Ad‐MITOL‐shRNA. Furthermore, the activity of SOD and GSH‐Px was upregulated while myocardial MDA level was reduced following irisin treatment, the effect of which was reversed via Ad‐MITOL‐shRNA‐mediated MITOL inhibition. Thus, these data illustrated that irisin‐mediated protection against MI/R‐induced apoptosis and oxidative stress was diminished through MITOL inhibition.

### 3.7 MITOL participates in the protective effects of irisin against SI/R injury via ROS generation and cell apoptosis

**FIGURE 5:**
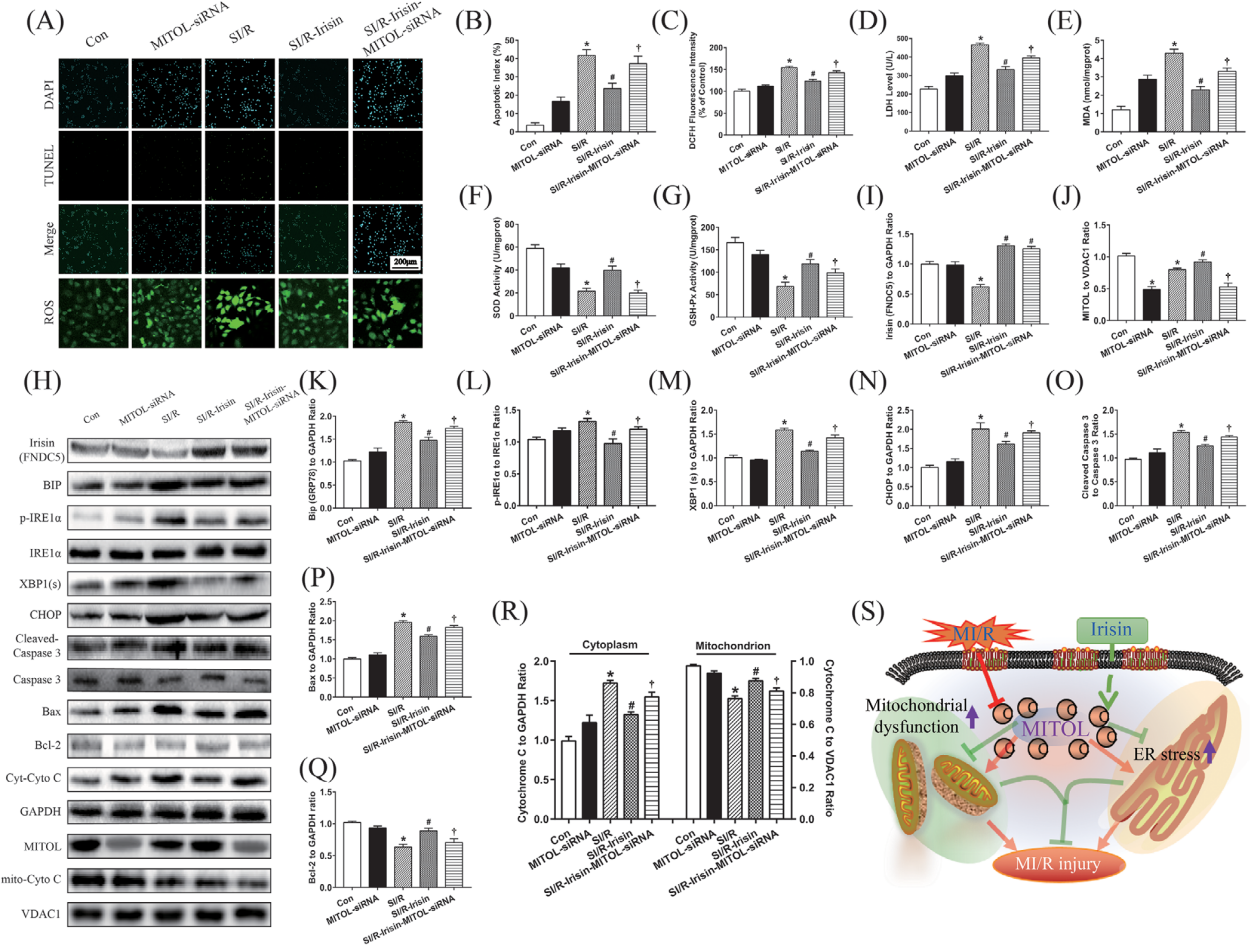
Irisin protected H9c2 cells against SI/R injury by alleviating ER stress and reducing apoptosis via the MITOL signaling pathway. A, Representative images of apoptotic cardiomyocytes and ROS production of H9c2 cells with TUNEL staining and DCFH‐DA (green). TUNEL: green fluorescence presents TUNEL‐positive nuclei; DAPI: blue fluorescence presents the total nuclei of cardiomyocytes. B, Cell apoptosis presented as the apoptotic index (×100%). C, Intracellular ROS levels were examined by DCFH‐DA fluorescence. D, LDH level. E, Cellular MDA content. F, Cellular SOD activity. G, GSH‐Px activity. H, Representative protein images by western blot from different groups. I, Irisin level with or without irisin and MITOL siRNA treatments. J, Expression of MITOL. K‐N, Expression of ER stress related proteins. O‐R, Expression of apoptosis related proteins. S, Schematic diagram showing the roles of MITOL, mitochondrial dysfunction, and ER stress in cardiac function in response to MI/R injury. MI/R injury significantly reduced MITOL expression, aggravated ER stress, increased cellular apoptosis, thus ultimately impairing cardiac function. All these effects were reversed by irisin treatment via MITOL upregulation. I, Con: cells were cultured in serum‐free DMEM medium. (II) MITOL‐siRNA: cells were pretreated with MITOL siRNA for 24 h and cultured in DMEM medium. (III) SI/R: cells were exposed to ischemic buffer for 50 mins and then returned to normal DMEM for 4 h. (IV) SI/R‐Irisin: cells were pretreated with irisin for 4 h and then exposed to the ischemic buffer for 50 min followed by incubation in serum‐free DMEM for 4 h. (V) SI/R‐Irisin‐MITOL‐siRNA: cells were pretreated with MITOL siRNA for 24 h and irisin for 4 h then exposed to the ischemic buffer for 50 min followed by incubation in serum‐free DMEM for 4 h. The results are presented as mean ± SEM, n = 3 in each group. ^*^ *P* < 0.05 versus the Con group, ^#^ *P* < .05 versus the SI/R group, **^†^** *P* < 0.05 versus the SI/R‐Irisin group

To discern whether irisin protects against MI/R injury and ER stress through modulating MITOL activity, H9c2 cells were employed to develop a SI/R cell culture model. Similar to in vivo study, cells in the control and SI/R groups were treated with various concentrations of irisin. Our data revealed that 5 ng/mL irisin incubation for 2 h displayed a pronounced protective effect against SI/R injury against ER stress. As shown in Figure 5A,B, SI/R procedure overtly promoted apoptosis. Inhibition of MITOL enhanced the number of apoptotic cells, the effect of which was alleviated by irisin administration. Furthermore, SI/R procedure significantly increased ROS production (Figure 5A,C), LDH (Figure 5D), and MDA content (Figure 5E) while inhibiting SOD activity (Figure 5F). Inhibition of MITOL using MITOL siRNA produced similar effects to that of SI/R injury but to a lesser degree, the effect of which was reversed by irisin treatment. These findings suggested that irisin treatment attenuated SI/R injury and ER stress‐induced apoptosis provoked by MITOL inhibition.

### 3.8 Irisin protects H9c2 cells against SI/R by alleviating ER stress and reducing apoptotic protein expression through upregulation of MITOL

Next, we determined the effects of irisin on apoptotic and ER stress proteins in H9c2 cells. As shown in Figure 5H‐R, levels of ER stress markers GRP78, p‐IRE1α, XBP1(s), and CHOP, as well as apoptotic proteins Bax, cytosolic Cytochrome C, and cleaved Caspase‐3 were upregulated while Bcl‐2 was downregulated in response to SI/R. To elucidate the mechanisms underlying the protective role of MITOL against ER stress, we examined the effects of MITOL ablation using siRNA. As expected, MITOL siRNA treatment upregulated levels of GRP78, p‐IRE1α, IRE1α, XBP1(s), and CHOP. Taken together, these observations indicated that inhibition of MITOL provoked ER stress‐induced apoptosis in H9c2 cells, and irisin treatment significantly reduced ER stress and ER stress‐induced apoptosis triggered by MITOL inhibition or SI/R.

## 4 DISCUSSION

Our results confirm that irisin protected cardiomyocytes against MI/R injury and alleviated ER stress and mitochondrial damage through activation of MITOL. In this study, pretreatment mouse hearts with irisin prior to MI/R challenge protects cardiomyocytes against I/R‐induced injury, as reflected by increase of cell viability and improvement of cardiac function, and reduction of myocardial infarct size, ER stress, and cell apoptosis. Inhibition of MITOL expression significantly compromised the protective effect of irisin on MI/R injury, indicating that irisin offers its cardioprotection partially through activation of MITOL.

As a novel myokine, irisin has shown benefits in a number of cardiac pathologies.^21^ Irisin protected the heart against I/R injury and increased cell survival by improving mitochondrial function as evidenced by upregulation of mitochondrial ETC complexes and reducing myocardial apoptosis.^14^, ^15^ The precise molecular mechanisms were further demonstrated that irisin upregulated SOD‐1 expression and p38 phosphorylation, inhibited the mPTP opening to alleviate mitochondrial dysfunction.^14^ It was shown that irsin interacted with SOD2 and restored the mitochondrial localization of SOD2 to ameliorate oxidative stress in response to I/R injury.^23^ Our results further showed that irisin reduced the MDA level of myocardium and cardiomyocytes and inhibited ROS generation by enhancing the antioxidant enzyme GSH‐Px activity to reduce I/R‐induced mitochondrial damage. Moreover, previous studies showed that ER stress played a major role in myocardial ischemia injury and irisin displayed the protection of macrophages from oxidized low‐density lipoprotein‐induced apoptosis via inhibiting ER stress signal pathway.^19^ However, our data for the first time showed that irisin pretreatment downregulated the levels of Bip, p‐IRE1α, XBP1(s), and CHOP in the face of MI/R injury, thus reducing MI/R‐induced ER stress in vivo. These effects further resulted in the reduction of cell apoptosis as evidenced by reduction of Caspase 3, Cyto C, and Bax, and the increase of Bcl‐2. These observations led us to believe that irisin plays a protective role against MI/R injury by alleviating ER stress and mitochondrial damage.

MITOL is a mitochondrial ubiquitin ligase and promoted the ubiquitylation of ER stress sensor IRE1α to alleviate ER stress‐induced apoptosis.^28^ On the other hand, MITOL that was localized in mitochondria regulated mitochondrial fission and reduced injured mitochondria‐induced oxidative stress, which played an important role in maintaining mitochondrial function and cell survival.^38^, ^44^ Further evidence demonstrated that MITOL directly interacted with and ubiquitinated Mfn2 to enhance ER tethering to mitochondria.^39^ Therefore, these results clearly showed that MITOL was a key partner to regulate the interplay between ER and mitochondria at their contact site. However, the roles of MITOL in mouse hearts suffering from MI/R injury were not fully elucidated. Our results presented here showed that MI/R injury remarkably inhibited MITOL expression and increased levels of Bip, p‐IRE1α, XBP1 (s), and CHOP as well as apoptotic proteins Caspase 3 and Bax (while decreasing the anti‐apoptotic Bcl‐2). This is supported by TUNEL staining where ER stress and apoptotic rate were significantly increased in response to MI/R procedure. Further evidence from our study demonstrated that inhibition of MITOL enhanced ER stress and myocardial apoptosis in comparison with MI/R‐irisin group in vivo. Our in vitro study also revealed that MITOL siRNA markedly increased cell apoptosis and ER stress. These findings denoted an important role for diminished MITOL in ER stress and apoptosis during MI/R. Furthermore, pretreatment with irisin prior to MI/R normalized MITOL expression following IR and improved mitochondrial function and decreased ER stress. Inhibition of MITOL using siRNA and MITOL shRNA partially dampened the protective effect of irisin on MI/R‐induced apoptosis and ER stress, denoting a vital role for MITOL in irisin‐offered cardioprotection. Earlier evidence has indicated that MITOL participated in the recruitment of Parkin in mitochondrial localization, whereas MITOL knockdown weakens the ubiquitylation of PINK1/Parkin.^35^ In addition, MITOL attenuates cytotoxicity of ROS and promotes its degradation.^36^ Moreover, MITOL regulates mitochondrial dynamics including mitochondrial morphology and transport.^45^ Furthermore, MITOL was shown to be responsible for hypoxia‐induced MFN2 degradation in HDAC6 deficient cells.^37^ Our data showed that the Cyto C expression and ROS production were remarkably increased following MI/R injury. While irisin treatment decreased levels of ROS and Cyto C, these irisin‐offered responses were attenuated by MITOL inhibition in MI/R injury. This notion received support from our in vitro experiment where MITOL inhibition aggravated SI/R injury by offsetting irisin‐induced protective effect in cardiomyocytes. However, the exact receptor of irisin involved in this study is not verified in our study and will be investigated in the further study. Taken together, our results indicate that irisin protects the heart against MI/R or SI/R injury partially by inhibiting ER stress and cellular apoptosis via activating MITOL. Thus, MITOL was shown to participate in the pathogenesis of MI/R injury, which inhibited ER stress and mitochondrial dysfunction, thus reduction of cardiomyocyte loss and preserving cardiac function in our study.

## 5 CONCLUSIONS

In conclusion, major findings from our present study revealed that irisin pretreatment protects the heart against MI/R injury, ER stress, mitochondrial damage, ROS generation, and apoptosis through MITOL activation. These results should shed some lights towards targeting irisin and MITOL as potential therapeutic strategies in the management of MI/R in patients with STEMI. Administration of irisin is expected to protect against MI/R‐induced cardiac dysfunction via MITOL‐mediated ER stress and apoptosis inhibition, thus ultimately preserving cardiac function (Figure 5). Our data support a favorable role for irisin treatment and MITOL activation as therapeutic avenues for MI/R‐induced cardiac dysfunction. Targeting MITOL and irisin administration may offer therapeutic promises in MI/R‐induced injury, although further studies were required for clinical application.

## CONFLICT OF INTEREST

The authors declare that they have no conflict of interest.

## Supporting information

Supporting Information

Supporting Information

## Data Availability

The data used in the present study are available from the corresponding author on reasonable request.
