# Supplementary figures and images for: Irisin attenuates myocardial ischemia/reperfusion‐induced cardiac dysfunction by regulating ER‐mitochondria interaction through a mitochondrial ubiquitin ligase‐dependent mechanism
Source: Clin Transl Med. 2020 Sep 14;10(5):e166. doi: 10.1002/ctm2.166 (PMC7507588; doi:10.1002/ctm2.166)

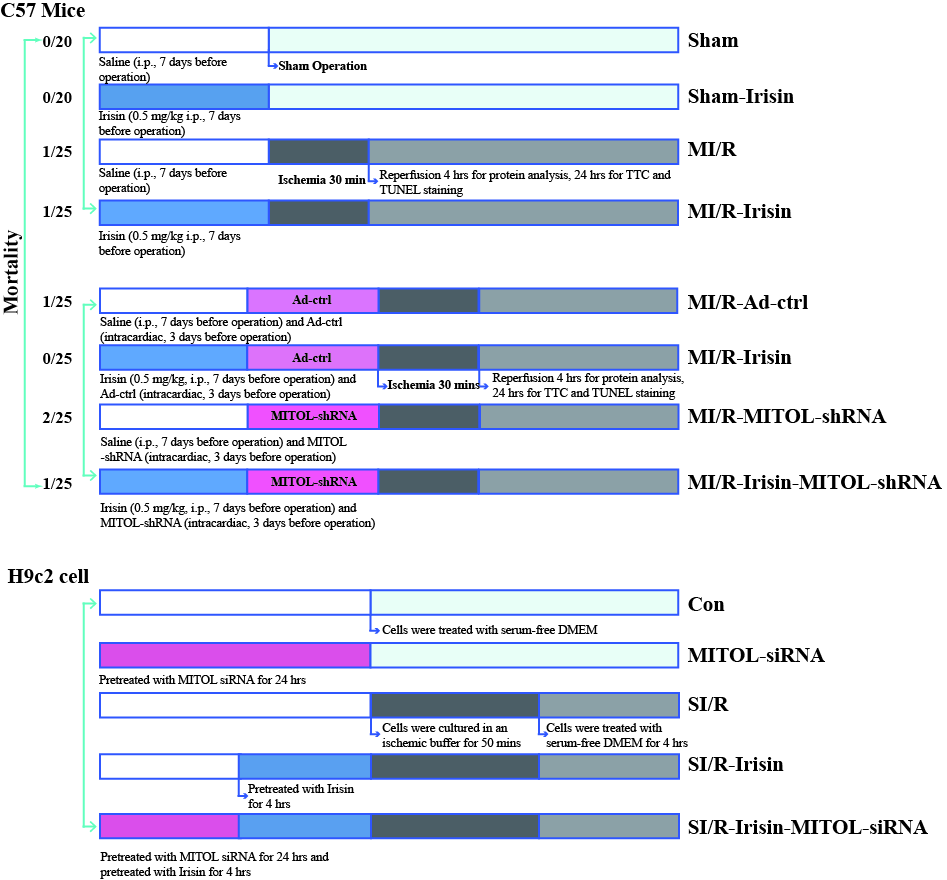

Supplement: Supplementary file 2 — Supporting Information [file CTM2-10-e166-s002.tif]
