# Supplementary material for: Irisin attenuates myocardial ischemia/reperfusion‐induced cardiac dysfunction by regulating ER‐mitochondria interaction through a mitochondrial ubiquitin ligase‐dependent mechanism
Source: Clin Transl Med. 2020 Sep 14;10(5):e166. doi: 10.1002/ctm2.166 (PMC7507588; doi:10.1002/ctm2.166)
Supplement: Supplementary file 1 — Supporting Information [file CTM2-10-e166-s001.docx]

**FIGURE LEGENDS**

Fig. S1 The protocol of *in vivo* and *in vitro* experiments and the mortality for single group.

*In vivo*: In the Sham, MI/R, MI/R-Ad-ctrl, and MI/R-MITOL-shRNA group, mice were treatment with 7 μL saline (i.p.) for 7 days. In the Sham-Irisin, MI/R-Irisin, MI/R-Irisin-MITOL-shRNA group, mice were treatment with recombinant irisin (0.5 mg/kg, i.p.) for 7 days. The ad-ctrl or MITOL-shRNA were administered at a concentration of 1×10^11^ PFU/mL by intracardiac injection 3 days before MI/R surgery. *In vitro*: In the SI/R-Irisin, and SI/R-Irisin-MITOL-siRNA groups, H9c2 cells were pre-treatment with 5 ng/ml irisin for 4 hrs. Cells were cultured in an ischemic buffer for 50 min and then treated with surum-free DMEM for 4 hrs in the SI/R procedure. Sham: mice underwent sham operation. Sham-Irisin: mice were pretreated with recombinant irisin underwent sham operation. MI/R: MI/R surgery was performed in this group. MI/R-Irisin: mice were pretreated with recombinant irisin then subjected into MI/R surgery.
